# Autism and psychopathology - prevalence, identification, and symptoms equivalence: study protocol

**DOI:** 10.3389/fpsyt.2024.1447262

**Published:** 2024-08-28

**Authors:** Marco O. Bertelli, Annamaria Bianco, Shoumitro Deb, Daniela Scuticchio, Shaniko Kaleci, Maria Luisa Scattoni

**Affiliations:** ^1^ CREA (Research and Clinical Centre), Fondazione San Sebastiano, Misericordia di Firenze, Florence, Italy; ^2^ SIDiN, Italian Society for Neurodevelopmental Disorders, Florence, Italy; ^3^ Imperial College London, London, United Kingdom; ^4^ Department of Surgical, Medical, Dental and Morphological Sciences with Interest in Transplant, Oncology and Regenerative Medicine, University of Modena and Reggio Emilia, Modena, Italy; ^5^ Research Coordination and Support Service, Istituto Superiore di Sanità, Rome, Italy

**Keywords:** Autism spectrum disorder, intellectual disabilities, psychopathology, psychiatric disorders, prevalence

## Abstract

**Objective:**

Despite increasing evidence of high psychopathological vulnerability in people with Autism Spectrum Disorder (ASD) and/or Intellectual disability (ID), comprehensive data on prevalence and presentation of psychiatric disorders (PD) in people with significant cognitive and communication impairment are lacking. The extent to which PD can present with behavioral/observable symptoms and include Problem Behaviors (PB) has also been scarcely evaluated through population-based studies. The paper presents the protocol of a cross-sectional study aimed at filling these gaps, referred to a large multicentric Italian population-based sample of adolescents and adults.

**Methods:**

A battery of validated scales, SPAIDD, DASH-II, DiBAS-R, and STA-DI, is used to support and control for clinical diagnoses of PD. Study population is stratified according to different independent variables such as the severity of ID and ASD, gender, age group, and source of recruitment. A network analysis will be carried out to identify the most central behavioral symptoms for the various PD and their relationship with PB. Overlap between psychiatric symptoms and ASD and ID phenotypes is also addressed.

**Results and Conclusion:**

This study should provide valuable insight into better diagnostic accuracy, leading to well-informed interventions to improve the quality of life of people with ASD and/or ID.

## Introduction

Recent research confirms that problem behaviors (PB) and disruptive/impulse-control/conduct disorders are common in people with Autism Spectrum Disorder (ASD) and/or Intellectual Disability (ID) and represent the greatest obstacles to clinical, rehabilitation and social integration interventions (1–4).

About half of the adults with ID and a similar proportion with ASD receive psychotropic medications (5, 6), mostly antipsychotics (7, 8), despite the guidelines of the last three decades indicating a weighted use (9, 10). In most cases, antipsychotics are used to manage PB in the absence of a serious mental illness. Similar trends were noted for antidepressants, mood stabilizers and sedatives (11). This off-licence use of psychotropics is a major public health concern and, in some cases, may raise ethical dilemmas (12).

The available studies report a high rate of one or more psychiatric co-occurring conditions in adolescents and adults with ASD/ID that compromise the person’s adaptive skills and quality of life (13). In a recent metanalysis, Micai and colleagues (14) found developmental coordination disorder (87%), sleep-wake problem (43%), ADHD (37%), anxiety disorder (35%), ID (33%), feeding and eating disorder (32%), and disruptive behavior (28%) to be the most common co-occurring conditions in children and adults with ASD.

In a previous meta-analysis, Lai and collaborators (15) found overall pooled prevalence estimates of 28% for ADHD, 20% for anxiety disorders, 13% for sleep-wake disorders, 12% for disruptive, impulse-control, and conduct disorders, 11% for depressive disorders, 9% for obsessive-compulsive disorder, 5% for bipolar disorders, and 4% for schizophrenia spectrum disorders in people with ASD. Older age was associated with a lower rate of ADHD, sleep-wake problems, feeding and eating disorders and a higher rate of depressive, bipolar, and schizophrenia spectrum disorders than younger age. Among autistic adults, another recent meta-analysis showed that the most common psychiatric comorbidities were ADHD (25.7%) followed by mood (18.8%) and anxiety disorders (17.8%) (16).

Simonoff and colleagues (17) conducted a population-derived cohort study on a group of 112 ten- to 14-year-old children with ASD, showing that one comorbid disorder was observed in 70% of the sample and two or more in 41%. The most reported comorbidities were social anxiety disorder (29.2%), ADHD (28.2%), and oppositional defiant disorder (28.1%).

For adults with ID, reported prevalence of psychopathology ranges from 14.5% (excluding PB, ADHD, ASD, dementia, and personality disorder, people aged 65 and over, and people with severe ID) (18) to 43.8% (adults with moderate to profound ID only) (19), depending on the sample and the assessment used (18–22). The largest adult population-based prevalence study in which each person (aged 16 years and over) was individually assessed included 1023 adults with ID (21). This study reported a point prevalence of Psychiatric Disorders (PD) ranging from 22.4% using clinician’s diagnosis to 13.9% using DSM-IV-TR criteria (or 40.9% if PB are also included). It reported rates separately for adults with mild ID at 25.4% and for adults with moderate to profound ID at 30.2% (21).

The effect of the severity of ID on the prevalence of PD is controversial, with some findings indicating higher rates in persons with severe/profound ID (23–25) but others indicating the opposite (26, 27). Some differences are accounted for by whether or not PB and/or ASD were included within the definition of PD, as they occur more commonly in people with more severe ID.

The presence of ID or borderline intellectual functioning (BIF) is common among people with ASD as well as the presence of major communication difficulties. Some studies report that around 65% of people with ASD also present ID or BIF (28, 29). However, few studies were conducted to explore the prevalence of PD in people with co-occurrent ASD and ID. The recent studies reported prevalence rates ranging from 14% to 94% on children (30–32) and from 1% to 41% on adults (33–35).

In people with ASD, the prevalence of psychiatric morbidity seems to increase when supports need is higher and/or ID co-occurs (13, 15, 36–38). However, other studies reported small (39) or no difference (40) in the rate of psychopathology in people with ASD with and without co-occurrent ID.

Mood disorders (41), anxiety disorders (42), schizophrenia spectrum disorders, and impulse control disorders (43–45) are the most frequent psychiatric conditions reported in people with ASD and ID. Teens with co-occurrent ASD and ID seem to present increased rates of inattention, hyperactivity, and impulsivity compared with people with ID alone (46). Furthermore, anxiety, mood, sleep problems, organic syndrome, stereotypies, and tics are also more prevalent among people with both ID and ASD (37).

There is an overlap between the ASD symptoms and symptoms of many PD, which may be particularly confusing to clinicians without specific expertise in ASD psychopathology. Murphy and colleagues analyzed a series of 859 adults, divided into two groups with respect to the ability of the basic symptomatology to satisfy or not the ICD-10 diagnostic criteria for ASD (47). In these groups, the rate of PD exceeded that of the general population in both number and severity. However, the prevalence of anxiety disorders and obsessive-compulsive disorder was much higher in the group meeting the ICD-10 diagnostic criteria of ASD than those who did not. Given the high rate of anxiety in the ASD population and the overlap between anxiety and obsessive-compulsive disorder symptoms, the true estimate of the rate of obsessive-compulsive disorder in this population may be hard to determine accurately. Similarly, the overlap between the symptoms of some forms of non-affective psychotic disorder and bipolar spectrum disorders requires further research (48).

Interpretation of published data is difficult as different studies used different methods, type of prevalence (point or lifetime), sample selection (including severe and profound ID or not) and size, diagnostic criteria, and tools to determine the presence of psychopathology in people with co-occurrent ASD and ID.

It’s also still unclear how prevalence can be affected by sex. While the majority of research (20, 26, 49, 50) showed no differences, some (51) indicated a considerably greater incidence in males than in females. As for general prevalence, some of these discrepancies may be explained by whether or not the definition of PD included PB and/or the co-occurrence of ASD and ID.

Reliability of prevalence rates across studies is particularly challenging in people with major cognitive and communication difficulties as they often cannot express their thoughts and feelings appropriately. As a result, the symptoms of PD manifest differently than in the general population (52) and adapted diagnostic criteria and tools are needed.

The reliability of the information gathered from many people with ASD and ID to arrive at a psychiatric diagnosis is often questionable because of their poor verbal skills, a tendency to acquiescence and, even for certain peculiarities in the experiential range, deviations from the norm with respect to attribution of meaning to the communicative contents. Some individuals with ASD and ID may present major difficulties to introspect, define their own experiences and communicate their discomfort or suffering. In many cases, particularly in people with severe and profound ID, the symptoms of PD may manifest through changes in behavior from the baseline (52). The term ‘behavioral equivalent’ is used by some to explain this possibility of psychiatric symptoms manifested through behavior changes (52, 53). Others found no evidence to support the concept of ‘behavioral equivalent.’ Some hypothesized that PB are part of an emotional dysregulation spectrum (54, 55).

Information from family members or professional care providers, about the person’s mental state can be unreliable and contradictory. Sometimes, the psychiatric symptoms are presumed to be part of the ID phenotype, leading to a false negative psychiatric diagnosis. This phenomenon is described as ‘diagnostic overshadowing’ (56).

As discussed in the previous sections, the psychopathology in adults with co-occurrent ASD and ID, particularly among those with major cognitive and communicative difficulties, has not been studied properly. Therefore, in the current study, we aim to explore psychopathology among adolescents and adults with co-occurrent ID and ASD in more detail to find out the differences in the manifestation of psychiatric symptoms in this population compared with the general population. Furthermore, we will explore the overlap between PB, ASD/ID phenotypes, and PD.

## Study design

The proposed study is an observational, analytical, transversal, multicenter study aimed at detecting the prevalence and clinical presentation of PD in people with ASD and in people with ID presenting considerable cognitive and communication difficulties.

## Reference population

The population included is represented by men and women with ASD and/or ID aged between 16 and 90 years. This age range is consistent with that taken into account in the validation trials of the SPAIDD-G (57), which is used as the primary psychopathology assessment instrument in the present study.

Participants are recruited from various settings, including small and large community residential homes with residents of different abilities, rehabilitation centers, medical clinics, and family homes.

The sample is stratified based on the ASD three support need levels indicated by the DSM-5, the severity of the possibly co-occurring ID (borderline, mild, moderate, severe, and profound), the type of recruitment center, and some background variables.

Because of the lack of resources and the need to recruit as many participants as possible, no limit and no randomization have been considered for the inclusion of recruitment centers. Consequently, no preliminary check has been done for the adequate spread of urban vs rural and affluent vs deprived communities. The background characteristics of recruitment centers will be assessed afterwards using statistical stratification during data analysis. The coordinating center has produced a call to participate in the study and disseminated it through various media and scientific congresses. All the potential recruiting centers that will contact the coordinating center will be checked for adequacy in terms of the possibility of providing all the background information and carrying out all the assessments requested by the study protocol. A specific checklist was produced to send out to all centers showing an interest in recruiting participants.

All recruiting centers are asked to enroll study participants according to the following criteria: consecutive access to the center, one female to every four males for those with a diagnosis of ASD, and one female to every two males for those with a diagnosis of ASD and ID or ID alone.

## Main objectives

The main objective of the study is to determine the prevalence rates of PD in people with ASD alone, ID alone, ASD and BIF, and ASD and ID. The sample is further stratified according to the different levels of support needed for participants with ASD and different levels of ID severity for participants with ID, characteristics of the recruitment centers and personal background.

Secondary objectives are: 1) to identify different manifestations of psychiatric symptoms in the participants compared with the general population; 2) to investigate the relationship between PB and PD, both generally and specifically; 3) to point out clinical features that might enhance the ability to differentiate between ASD, ID, and co-occurring PD.

## Randomization

A centralized and automated randomization procedure is adopted. To perform it, an easy-to-manage, protected database is used, in which all cases enrolled by recruitment centers are put together and preliminarily coded. Specific databases are built to ensure data integrity from every single recruitment center. Further specific randomization lists are also provided for the different stratification areas described above. Within age, gender, ID severity, and support need subgroups, every second person will be randomly selected.

Strata are based on the combination of setting (e.g., urban vs. rural or congregate setting vs. independent living), age, gender, ID severity, and support need at enrolment. Explorative subgroup analyses will be carried out, with subgroups identified by strata used in the randomization or other clinical parameters. Odds Ratios (ORs) and 95% Confidence Intervals (CIs) will be calculated and graphed through forest plots.

Results obtained from the randomized sample are compared with those of the full sample in order to evaluate the impact of biases in the sample gathering.

Before the research started and the protocol was submitted to the local ethical committee, a preliminary informal survey on the interest to participate in the study was undertaken among clinical staff and referring clinicians of various mental health care providers, self-advocacy groups, and family associations.

## Blindness (masking)

In order to increase the precision of the psychiatric diagnoses of study participants, screening and psychopathological diagnostic tools (see below) are administered by trained research staff members blindly to the clinical diagnoses already made by clinicians in medical records. Results of instrumental assessment and clinical diagnosis are compared and discussed afterwards by the whole clinical and assessment staff in order to produce a final diagnosis that was the most reliable with the real clinical condition of the study participant.

This seems a useful procedure to follow in light of the fact that while raters of the instrumental assessment of all recruitment centers underwent specific training and evaluation of inter-rater reliability, this was not possible for clinicians with patients in charge.

## Information gathering

In each recruitment center, for all participants identified based on the aforementioned inclusion criteria, the following data are collected, derived from the consultation of the clinical and/or administrative documentation: date of birth, gender, type of residence, diagnosis of ASD (with the level of support needed) and ID (with the level of severity), diagnosis of co-occurring PD, presence of PB (possible instrumental scoring), presence of significant physical disorders, including epilepsy and other neurological disorders. A predefined proforma is used to standardize and facilitate the collection of the data completed by the professionals in each center. All information about the study participants has been handled and transmitted anonymously, as stated below. Subsequently, the data relating to the research object have been collected by research staff members through the tools and procedures detailed below.

## Sample size estimate

**Figure 1:**
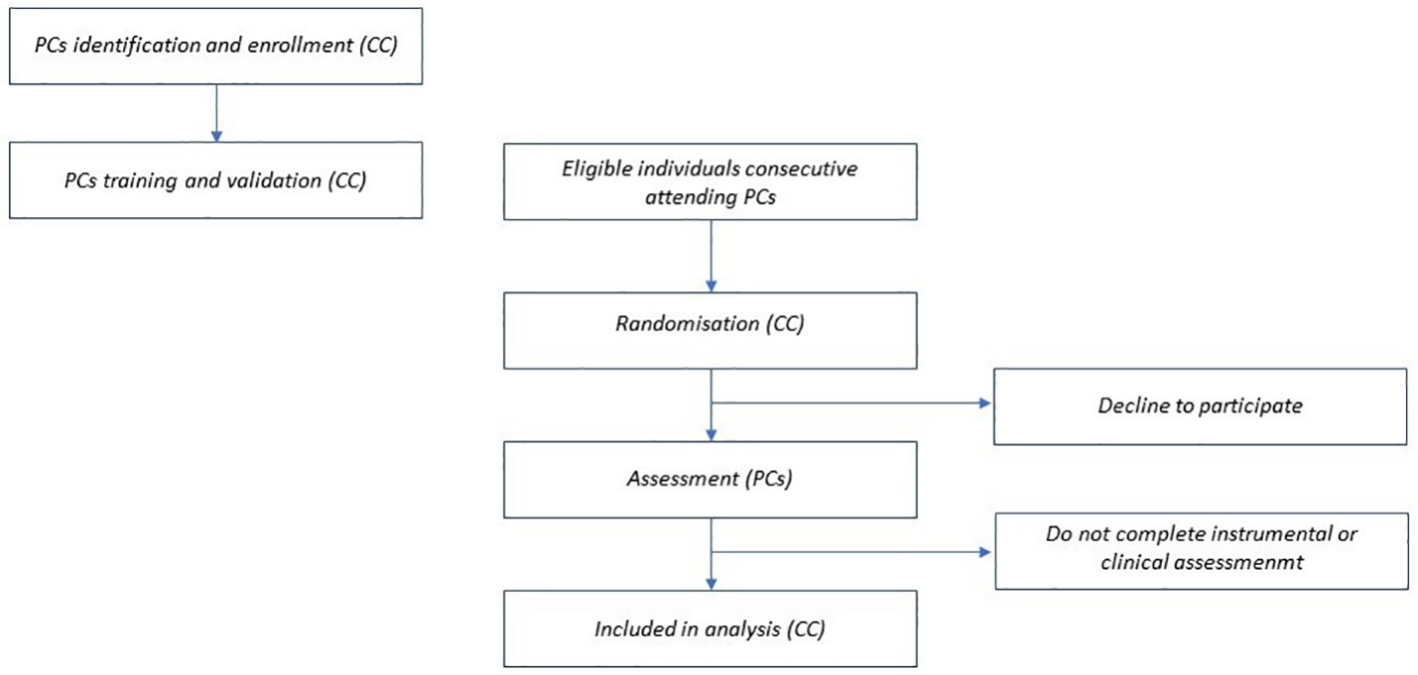
Flow chart of the study participants recruitment. PCs, participating centers; CC, coordinating center.

Based on the clinical and research experience of the proponents and the literature review, the percentage prevalence of PD in persons with ASD is around 70 (58), and in persons with ID is around 40 (13), and even higher in persons with ASD and ID (13). Considering a 95% confidence interval and a desired absolute precision of 4.5%, the minimum sample size to be representative of the research population is about 480 participants. To obtain this number after randomization, starting from a general sample of approximately 960 cases is necessary. Randomized participants who will not either consent or participate for several reasons (a rough guide is 10-25%) will be randomly replaced among those excluded by the first randomization. The flow chart of the study participants recruitment is reported in **Figure 1**.

## Tools

For the assessment of the full range of PD, the following tools are used:

### Systematic Psychopathological Assessment for persons with Intellectual and Developmental Disabilities – General screening

The SPAIDD-G is part of a wide tools system to support professionals working with persons with ID/ASD in the identification of psychopathological symptoms and syndromes (57). It includes 56 items, which represent descriptions of the most frequent observable and behavioral aspects of all the symptoms that appear in the various DSM-5 diagnostic categories. These items were created so that they could be evaluated by a mental health professional using data obtained from interviews with a family member of the person with ID/ASD or any other information who has a good sense of changes in the behavior of the people they care for. Raters do not have to attribute or rate the severity of any score, they only have to indicate the presence or absence of an item by ticking the appropriate box. Items are presented randomly and not by diagnostic subscales. The SPAIDD-G evaluates the following syndromic groupings: nutrition/feeding disorders, psychotic disorders, mood disorder-depression, mood disorder – mania, anxiety disorders, side effects of drugs, delirium, dementia, substance-related disorders, odd personality disorder, dramatic personality disorder, anxiety personality disorder, impulse control disorder, ASD, dissociative identity disorder, somatic symptom disorder, sexuality disorder and obsessive-compulsive disorder. The SPAIDD-G showed very good psychometric characteristics (57, 59).

For the present study, two new subscales for ADHD and Trauma and stressors-related disorders have been added. The symptoms of the former have been derived from those recently proposed by the Royal College of Psychiatrists (60), while symptoms of Trauma and stressors-related disorders have been elaborated based on those of the Diagnostic Manual – Intellectual Disability 2 (61).

### Diagnostic assessment for the severely handicapped

The DASH-II is an internationally well-known structured informant interview assessing the presence of PD and maladaptive behavioral symptoms in people with severe cognitive and communication impairments (62–67).

SPAIDD and DASH-II are used to detect problem behaviors.

For the assessment of mood and psychotic disorders, the specific tools of the Systematic Psychopathological Assessment for persons with Intellectual and Developmental Disabilities have been used (57, 68–70). See **Table 1** for details.

**Table 1:** APPrISE assessment tools for specific psychopathological conditions and ASD.

TOOL	DESCRIPTION	PSYCHOMETRIC CHARACTERISTICS
Systematic Psychopathological Assessment for persons with Intellectual and Developmental Disabilities - Mood Disorders (SPAIDD-M) (68, 69).	Part of the SPAIDD battery. It includes 66 items organized in three sections, for mood disorders symptoms, chronological and course specifiers, and duration and qualitative criteria.	- Internal consistency (Cronbach’s α): from.81 to.93 - Inter-rater reliability (Cohen’s K): from.87 to.57. - Concurrent validity (with DASH-II): 98% - Correlation with clinical diagnoses: (Pearson) rp=.053 and (Spearman) rs=.053, p=.000.
Systematic Psychopathological Assessment for persons with Intellectual and Developmental Disabilities - Psychotic Disorders (SPAIDD-P) (57, 70).	Part of the SPAIDD battery. It consists of 24 items, including all psychotic symptoms and chronological criteria for all the disorders within the group.	- Internal consistency (Cronbach α):.60 - reliability with clinical diagnoses.28 (p≤.01) - Three factors with Eigenvalues ≥2, (overall explaining 50% of variance): “hallucinatory”, “delusional” and “paranoid”.
Systematic Psychopathological Assessment for persons with Intellectual and Developmental Disabilities – Autism Spectrum Disorder (SPAIDD-ASD) (71).	Part of the SPAIDD battery. It includes 24 items, to be completed in a dichotomic way (presence or absence) by a rater based on information provided by the person with ID or an informant.	- Internal consistency (Cronbach’s α): 0.81 - Inter-rater reliability (Cohen’s K): 0,79 - Correlations with the Italian adaptation of the DASH-II and the STA-DI scores: (Pearson) rp=.055
Autism Rating Scale in People with Intellectual Disabilities (STA-DI) (72).	Is the Italian version of the Scale of Pervasive Developmental Disorder in Mentally Retarded Persons (PDD-MRS; 73), which is a screening instrument administered to the subject or to the caregiver useful to guide the clinician to identify ASD in people with ID.	- Internal consistency for persons in the norm group with functional speech (Cronbach’s α):.86; for those without speech,.81 - Inter-rater reliability (Pearson’s r):.83 - Stability of scores over a six-month period (Pearson’s r): from.81 to.86 - Stability over a 14-year period (Pearson’s r):.70.
Diagnostic Behavioral Assessment for ASD – Revised (DiBAS-R) (55, 74).		- Sensitivity: 83% - Specificity: 64% - Agreed with clinical diagnoses: 74% - Internal consistency (Cronbach’s α): 0.749.

To ascertain the validity of diagnoses of ASD in persons with ID the following tools have been used: Systematic Psychopathological Assessment for persons with Intellectual and Developmental Disabilities – Autism Spectrum Disorder (SPAIDD-ASD); Autism Rating Scale in People with Intellectual Disabilities (STA-DI); Diagnostic Behavioral Assessment for ASD – Revised (DiBAS-R). See **Table 1** for details.

## Clinical diagnoses

At the end of the study assessment procedure, all centers involved in the study are asked to retrieve from local clinicians and transmit to the coordinating center the eventual clinical diagnosis of co-occurring PD for all the participants enrolled. As above mentioned, diagnoses preceding the study assessment procedure were taken, when available, by consultation of the clinical and/or administrative documentation.

As far as authors’ knowledge there are no other validated tool validated in Italian that specifically address the full range of PD (DSM-5 based) in people with ASD and any degree of communication and cognitive impairment.

## Statistical analysis

All collected data have been drilled into an Excel database. The STATA17 and RStudio programs have also been used for data processing. Descriptive statistics are used to evaluate the clinical, background characteristics of the stratified sample and the primary objective. Continuous variables were presented as the number of patients (N), mean, standard deviation (SD), minimum (min), and maximum (max) and compared between subgroups using Unpaired Student’s t-test; Analysis of variance (ANOVA) is used to evaluate the differences of the SPAIDD under examination for variables with three or more categories, while categorical variables were presented as frequency (N, percentage [%]) and compared using Pearson’s chi‐squared test. For the secondary objective, the subgroup data have been compared with analysis of variance (ANOVA) and correlated with each other with the Pearson and Spearman tests. A block-wise and stepwise multiple regression is used to study the variance with respect to specific diagnoses, while the covariance and dependence between variables, including background ones, are calculated through hierarchical regression analysis. Cohen’s K is used to calculate the inter-rater reliability. Kappa is a measure of this difference, standardized to lie on a -1 to 1 scale, where 1 is a perfect agreement, 0 is exactly what would be expected by chance, and negative values indicate agreement less than chance, i.e., potential systematic disagreement between the observers. The interpretation of agreement adopted here is less than chance agreement (κ < 0), slight agreement (κ = 0.01 to 0.20), fair agreement (κ = 0.21 to 0.40), moderate agreement (κ = 0.41 to 0.60), substantial agreement (κ = 0.61 to 0.80), and almost perfect agreement (κ = 0.81 to 0.99). The interpretation of reproducibility adopted is marginal (κ = 0.00 to 0.40), good (κ = 0.40 to 0.75) and excellent (κ >0.75) (75).

To reduce the probability of errors due to multiple testing, authors will focus for psychopathological co-occurrences on results obtained with the main assessment tool, which is represented by the SPAIDD system. Furthermore, to control the fraction of false significant results among the significant results only, a false discovery rate approach will be carried out.

## Network analysis

Skewness and kurtosis are done to assess the distribution of all the SPAIDD battery tools, DASH-II, STA-DI, and DIBAS-R items network estimation is done using the Gaussian graphical model, graphical least absolute shrinkage and selection operator technique and extended Bayesian information criterion are used to establish the psychopathological behavioral equivalents network structure. These methods are used to shrink edges in the network and tune parameters to make the symptom network sparser and easier to interpret. R packages graph (version 1.9.4) and bootnet (version 1.5) are applied to visually estimate and illustrate the network model. Partial correlation analyses are done to build the association of each pairwise continuous variable (nodes) and form a network. The predictability, that is, the extent to which the variance of a node is explained by adjacent nodes in the network, is assessed using the R package MGM (version 1.2-13).

The robustness of the network structure is evaluated by estimating the accuracy of the edge weights by computing confidence intervals with a non-parametric bootstrapping method. Additionally, bootnet based on 1000 bootstraps is performed for each node to assess the stability of the centrality index.

After reviewing the network structure, the expected influence (EI) index is calculated to identify the most central symptoms for the various PD across categorical diagnoses. EI is a more appropriate measure of centrality to predict node influence on a network containing both positive and negative edges.

Furthermore, to explore bridge symptoms in the network that played essential roles in connecting two or more PD, the bridge EI (1-step) is calculated using the R package network tools (version 1.5.0). The centrality indexes of EI and bridge EI are reported as standardized values (Z scores) and considered stable when the correlation stability coefficient (CS-C) is larger than 0.25 and preferably larger than 0.50. A network comparison test (NCT) with 1000 bootstraps is done using the R package Network Comparison Test (version 2.2.1) by considering the moderating effects of gender, age, ASD severity, ID severity, and co-presence of ASD and ID.

## Training and information sharing

To optimize knowledge of the study design and inter-rater reliability of tools, an in-person meeting of all researchers involved in the study and a series of web conferences are conducted during the project’s initial phase by the coordinating center’s experts. At the end of this process, the successful acquisition of skills in the use of procedures and tools has been evaluated with standardized methods, including inter-rater reliability. Differences are discussed in case of low inter-rater reliability, and further training sessions are provided. Furthermore,the coordinating center provides a telephone number and two researchers to clarify all the queries of the researchers of the various participating centers.

The coordination center continues to provide information and clarification throughout the study. In addition, the coordination center has set up a website that provides regular updates on the study, addressed to researchers, participants, and their families.

## Feasibility

Participating centers must have at least one clinician with good training and long experience in psychopathological diagnosis and management of people with ASD and ID. This also refers to the diagnoses of PD and all the other clinical information. The utmost attention is paid to ensuring that the recruitment centers are geographically diverse.

The coordination center, which represented the core project group, has made available qualified personnel for the conduct of the multicenter study: 1. a psychiatrist expert in the research in the relevant area who is the principal investigator; 2. a group of researchers, both senior and junior, who are actively involved in the management of the activities required for the implementation and management of the study; two of them are also responsible for collecting data from the various participating centers; 3. experts who are responsible for preparing the form for electronic data entry and the centralized database for data collection and processing; 4. a statistician, already involved in the conduct of other studies in the sector; 5. a secretariat with many years of experience in managing the bureaucratic, ethical and administrative aspects of this type of research.

The coordination center has also made available all the tools and procedures for psychopathological evaluations.

Data management and the overall conduct of the study are overseen by a steering committee that includes representatives of the Italian Foundation for Autism (FIA) and the Italian Society for Neurodevelopmental Disorders (SIDiN). This committee is independent of the project management group and includes either a person with ASD or a family carer representative. FIA is an NGO that unites groups of caregivers of individuals with autism and developmental impairments, as well as scientific societies, private non-profit foundations, ethical bodies, and self-advocates. SIDiN is a scientific society, a special section of the Italian Society of Psychiatry.

## Good practice

The study was conducted in compliance with the guidelines for Good Clinical Practice (GCP -CPMP/ICH/135/95; G.U.R.I.n.191 of 18 August 1997). The alignment with these guidelines was checked by a specifically appointed person. There are randomized checks on the activities of some of the participating centers in the various phases of the study. The GCP rules are clarified to all the researchers involved during the constitution of the multicenter working group. Given the relatively simple and non-invasive nature of the assessment procedures, the only major risk possible was suboptimal enrolment. Should this have happened, remedial actions would have been implemented before the sample randomization procedure, also with the possible inclusion of additional or alternative recruitment centers.

In addition to observing the GCP protocol, the conduct of the experimentation and compliance with the general regulatory provisions have been constantly evaluated.

## Ethical aspects

The study received approval from the Regione Toscana Ethical Committee for Clinical Trials - Sezione Area Vasta Centro, on July 2, 2019, with the following registration code: APP_01 - APPriSE.

The study was conducted in accordance with the latest version of the Declaration of Helsinki (76). It does not in any way violate the individual ethical values of the subjects under observation and respects the principles of autonomy, benefit, and privacy of the participating subjects.

The study protocol has been submitted to the ethics committees of the participating centers and informed written consent to participate in the study has been requested from each participant or their legal representative. If a subject or their legally recognized representative is unable to read, an impartial witness must be present throughout the entire informed consent discussion.

All personal information is protected and anonymized through name coding and data custody systems, with access restricted to accredited researchers only. All the data transmitted as Excel files did not include any name nor any other sensitive data in order to overcome digital security issues.

The language used in the oral and written information concerning the study, including the written informed consent form, was as practical, non-technical as possible and understandable to the subject or his/her legally recognized representative and to the impartial witness, where applicable.

The initial total duration of the study is 24-29 months, divided into the following phases, some of which overlap: study planning and preparation (5 months), sample recruitment (20 months), assessment training (3 months), assessment and data collection (9 months), statistical elaboration (2 months), and report and paper production (3-6 months). Due to the COVID-19 pandemic, the study was extended for another 18 months.

## Discussion

As far as we know the APPrISE is the first study that specifically targets at assessing the prevalence of the full range of PD in adults with ASD, ID, and co-occurrent ASD and ID who present considerable cognitive and communication impairment that can significantly impact on the presentation of psychopathological symptoms. It is also the first study to include a network analysis.

A previous study similar to APPrISE was conducted by Bakken and collaborators (49) but it did not address the full range of psychopathology nor the reference to DSM-5 diagnostic criteria (or their adaption to ID, as indicated by DM-ID 2). Furthermore, the study by Bakken was specifically designed to compare the prevalence in people with ID and ASD versus ID alone, included a much smaller sample, relied on less complex recruitment and assessment procedures, and did not investigate the correspondence between instrumental screening and clinical diagnoses.

The largest adult population-based prevalence study by Cooper et al. (21) did not address PD co-occurring with ASD, neither alone nor in combination with ID as well as the relationship between PB and PD.

A recent meta-analysis found a very high heterogeneity in study designs aimed at detecting PD in our target population, due to all the major factors and biases mentioned above. Even the validity of any meta-analysis using pooled data is highly questionable. However, they showed prevalence rates being lower in population-based studies than in non-population-based studies and in low overall risk-of-bias studies as compared to the moderate overall risk-of-bias studies (26).

-Given the attention provided in the projecting in order to overcome many of the limits identified in previous research, the APPrISE study is expected to make a substantial new contribution to 1) gain knowledge on the prevalence and presentation of psychiatric co-occurrence in people with ASD/ID, especially adults; 2) identify clinical aspects capable of improving the capacity for differential diagnosis between ASD, ID, and co-occurring PD; and 3) improve personalized care of people with ASD/ID.

Co-occurring psychiatric conditions in ASD and ID are associated with the risk of poorer psychosocial and adaptive functioning, employment, and quality of life. Being aware of their prevalence in people with ASD/ID may be extremely informative for policymakers who should set appropriate service responses that can significantly improve the lives of people with ID and ASD, and their families.

## Data Availability

The original contributions presented in the study are included in the article/supplementary material. Further inquiries can be directed to the corresponding author.
